# The cryo-EM revolution: fueling the next phase

**DOI:** 10.1107/S2052252519000277

**Published:** 2019-01-01

**Authors:** Sriram Subramaniam

**Affiliations:** a University of British Columbia, Vancouver, BC V6T 1Z3, Canada; b Frederick National Laboratory for Cancer Research, Leidos Biomedical Research, Inc., Frederick, MD 21702, USA

**Keywords:** cryo-EM, national electron microscopy facilities, editorial

## Abstract

Cryo-EM has seen unabated growth and adoption in the last three years. Continued support at institutional, regional and national levels is essential to sustain its evolution.

In January 2016, exactly three years ago, Nature Methods featured cryo-electron microscopy (cryo-EM) as the ‘Method of the Year’ on its cover. The acknowledgment was not intended to imply that cryo-EM was a new method. Near-atomic resolution structures obtained using cryo-EM were already reported a decade ago. Microscopes capable of operation at cryogenic temperatures and generating images interpretable at atomic resolution were available two decades ago, and methods for analyzing low-dose cryo-EM images were being developed three decades ago. The most significant advance that made this revolution in structural biology possible was the development of direct electron detectors that ushered in the current era of electron counting and the recording of electron microscopic images as movies. By collecting a series of images instead of a single projection image, the motion of the proteins and specimen stage during the exposure could now be partially compensated. The integration of these detectors into electron microscopes suddenly made it possible to determine structures of proteins and protein complexes at unprecedented resolution.

If the access to these advanced tools and methods was restricted only to specialized practitioners of cryo-EM, this revolution in resolution would not have led to cryo-EM becoming the true revolution that it has, with the prospect of transforming all of structural biology, and perhaps cell biology too, in the years ahead. What is at the origin of this groundswell of unabated growth and adoption of cryo-EM that we have been witnessing in the last three years? There are at least three factors that are helping drive this next phase of the cryo-EM revolution.

First, the advances in streamlining many of the steps in the cryo-EM workflow have enabled non-specialists in adjacent, but closely related disciplines to take advantage of cryo-EM methods in their own research programs. The intricate steps involved in specimen preparation, data collection, data processing and interpretation have been broken down into sufficiently clear instructions that the barrier to entry is now lower than in previous years, especially at places where there is adequate availability of microscopes and the related accessories for specimen preparation and computing. The biggest beneficiaries at present have been X-ray crystallographers and NMR spectroscopists, but there is little doubt that this envelope will expand to include scientists in many other disciplines in modern biology.

Second, cryo-EM has transitioned from being a method that is only capable of handling a small subset of cryo-EM worthy specimens to a very large spectrum of proteins and protein complexes of broad general interest. This transition of cryo-EM from being a technology that was billed as a tool to analyze large and/or highly symmetric specimens, to one that can successfully tackle a range of proteins and protein complexes of broad general interest has been transformative. The structures of an impressive number of proteins, small and large, sometimes with extensive conformational spread, have been successfully analyzed by cryo-EM (see Fig. 1 ▸). Many of these protein complexes may never be coaxed to produce well ordered crystals for study by X-ray crystallography. It is important to recognize that in almost every instance, these selected successes in the application of cryo-EM rest on decades of advances in biochemistry, biophysics and protein science that laid the necessary groundwork. Nor can we overlook the fact that the landscape of macromolecular entities that are still intractable to analysis by cryo-EM remains immense. Yet, the future looks bright and there is every reason to hope that an ever-increasingly complex array of biological assemblies will be tackled by cryo-EM.

A third factor, perhaps a consequence of the first two, and with long-term impact, is the creation of national scale facilities that provide access to the latest cryo-EM technology. Modern electron microscopes are expensive, and the cost of their purchase and maintenance are often beyond the reach of many academic institutions. Besides, these are still finicky instruments; their mere purchase does not guarantee performance, and the expertise to get the best performance out of them is crucial, but not sufficiently widespread. Consequently, there is a very important role for federally funded facilities in ensuring adequate access (Stuart *et al.*, 2016 ▸). These facilities are necessary to supplement capacity at institutions that may have some infrastructure for cryo-EM, but even more so to those that do not have easy local access to data collection on advanced electron microscopes. The national center at Diamond Light Source (http://www.diamond.ac.uk/Science/Integrated-facilities/eBIC.html) has been exemplary in this regard, leading the way for democratizing access to cryo-EM technology. Many others such as the NeCEN center in the Netherlands (http://www.necen.nl), the Janelia Farm campus of the Howard Hughes Medical Institute (https://www.janelia.org/support-team/cryo-electron-microscopy) have also been instrumental in driving the adoption of cryo-EM by structural biologists. In the US, the first pilot national cryo-EM facility providing broad access was launched by the National Cancer Institute in 2017 (http://www.cancer.gov/research/resources/cryoem), and has already supported over 170 data collection sessions from 28 institutions across the US with just one Titan Krios microscope. The recent creation of three NIH-funded national centers (https://www.nih.gov/news-events/news-releases/nih-funds-three-national-cryo-em-service-centers-training-new-microscopists), each equipped with numerous modern electron microscopes, is certain to have a transformative impact by ensuring adequate access to cryo-EM instrumentation for US researchers.

The creation of synchrotrons catalyzed the dramatic growth of X-ray crystallography. National electron microscopy facilities will have a similar impact on the growth of cryo-EM. The emergence of cryo-EM as a powerful tool for structural biology led funders of biomedical research to ponder the question of the best model for enabling growth of this discipline, debating the relative merits of institutional, regional and national centers. The experience over the last three years teaches us that all three variants will be essential to sustain growth in the next phase of cryo-EM. Institutional investments are necessary to ensure adequate resources for training and providing access to screening instruments. Regional centers will facilitate concentration of expensive instrumentation, while also offering opportunities for on-site training and easy access. National centers, funded at the right scale will be necessary not just to function as user facilities, but also as hubs for innovation that help advance the frontier of the field, and take on important, long-term challenges of broad interest to the biomedical research community. It is clear that we need growth at all three levels. Maintaining a healthy equilibrium between institutional, regional and national resources will be essential to fuel the next phase of the cryo-EM revolution.

## Figures and Tables

**Figure 1:**
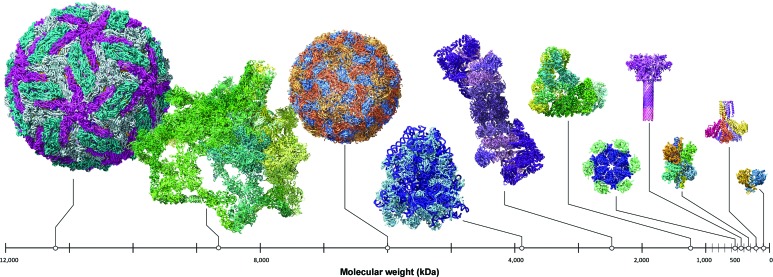
From large to small: the growing diversity of cryo-EM targets.
